# Effectiveness of workplace choice architecture modification for healthy eating and daily physical activity

**DOI:** 10.1186/s12889-024-18482-1

**Published:** 2024-04-01

**Authors:** Eeva Rantala, Saara Vanhatalo, Mikko Valtanen, Jaana Lindström, Jussi Pihlajamäki, Kaisa Poutanen, Pilvikki Absetz, Leila Karhunen

**Affiliations:** 1 Institute of Public Health and Clinical Nutrition, University of Eastern Finland, 70211 Kuopio, Finland; 2 Finnish Institute for Health and Welfare (THL), 00271 Helsinki, Finland; 3 VTT Technical Research Centre of Finland, 02044 Espoo, Finland; 4 Department of Mathematics and Statistics, University of Turku, 20014 Turku, Finland; 5 Department of Medicine, Endocrinology and Clinical Nutrition, Kuopio University Hospital, 70029 KYS Kuopio, Finland; 6 Faculty of Social Sciences, Tampere University, 33520 Tampere, Finland

**Keywords:** Choice architecture, Nudge, Workplace, Health promotion, Prevention, Type 2 diabetes, Behaviour change, Diet, Physical activity

## Abstract

**Background:**

Modifying the choice architecture of behavioural contexts can facilitate health behaviour change, but existing evidence builds mostly on small-scale interventions limited in duration, targets, strategies, and settings. We evaluated the effectiveness of a one-year hybrid type 2 implementation-effectiveness trial aimed at promoting healthy eating and daily physical activity with subtle modifications to the choice architecture of heterogeneous worksites. The intervention was contextualised to and integrated into the routine operations of each worksite. Effectiveness was evaluated in a quasi-experimental pre-post design.

**Methods:**

Intervention sites (*n* = 21) implemented a median of two (range 1–9) intervention strategies for healthy eating and one (range 1–5) for physical activity. Questionnaires pre (*n* = 1126) and post (*n* = 943) intervention surveyed employees’ behavioural patterns at work (food consumption: vegetables/roots, fruit/berries, nuts/almonds/seeds, sweet treats, fast food, water; physical activity: restorative movement, exercise equipment use, stair use). The post-intervention questionnaire also measured employees’ perception of and response to three intervention strategies: a packed lunch recipe campaign, a fruit crew-strategy, and movement prompts. Multi- and single-level regression models evaluated effectiveness, treating intervention as a continuous predictor formed of the site-specific dose (n intervention strategies employed) and mean quality (three-point rating per strategy halfway and at the end of the intervention) of implementation relevant to each outcome.

**Results:**

Multinomial logistic regression models found the intervention significantly associated with a favourable change in employees’ fruit and berry consumption (interaction effect of time and implementation *p* = 0.006) and with an unfavourable change in sweet treat consumption (*p* = 0.048). The evidence was strongest for the finding concerning fruit/berry consumption—an outcome that sites with greater dose and quality of implementation targeted by using strategies that reduced the physical effort required to have fruit/berries at work and by covering multiple eating-related contexts at the worksite. The quality of implementation was positively associated with the perception of (*p* = 0.044) and response to (*p* = 0.017) the packed lunch recipes, and with response to the fruit crew-strategy (*p* < 0.001).

**Conclusions:**

The results suggest that a contextualised, multicomponent choice architecture intervention can positively influence eating behaviour in diverse real-world settings over a one-year period, and that higher implementation quality can enhance intervention perception and response. However, outcomes may depend on the type of intervention strategies used and the extent of their delivery.

**Supplementary Information:**

The online version contains supplementary material available at 10.1186/s12889-024-18482-1.

## Background

The living environment can either help or hamper the adoption of healthy, sustainable lifestyles. A line of behavioural interventions pursues the former with focus on physical and social microenvironments. These interventions modify the way available options are presented in decision-making contexts to create choice architectures that gently “nudge” towards favourable behaviours without bans, substantial incentives, or rational argumentation [1, 2]. The approach acknowledges people’s sensitivity to contextual influences and tendency to invest little deliberation in many daily choices related to health [3]. The theoretical foundation lies in the dual-systems models that suggest behaviour to stem from the interaction of automatic and reflective cognitive processes, which are fallible and sometimes lead to unfortunate directions [4, 5].

Within the field of behaviour change research, choice architecture interventions mostly target the opportunity component of the COM-B system that defines three interacting conditions that are necessary for a behaviour to occur: capability, opportunity, and motivation [6]. Opportunity refers to the social and physical factors outside the individual that make a behaviour possible or prompt it [6]. Choice architecture interventions can influence behaviour directly via automatic processes or more indirectly via reflective processes that advance individual agency by facilitating deliberation on personal preferences, values, or goals [5, 7, 8]. The more direct, behaviourally oriented interventions typically reduce the physical effort required to engage in the desired behaviour [9, 10]. The more indirect, cognitively or affectively oriented interventions reduce cognitive effort, appeal to emotions, or support self-regulation, for example, with increased visibility or comprehensibility of behaviour-related information; with enhanced salience or attractiveness of preferred behaviours; with reminders or social reference points, or by facilitating commitment to beneficial actions [9, 10].

Efficacy trials conducted in controlled laboratory or field settings suggest that on average, choice architecture interventions promote behaviour change with small to medium effect sizes across behavioural domains; eating behaviour appearing particularly responsive to these interventions [8]. However, effects vary substantially across studies [8], and many trials have failed to demonstrate significant effects [11]. Simultaneously, scientific literature seems biased towards successful interventions with small sample sizes, creating overoptimistic expectations of intervention impact [12–14].

Workplaces provide an optimal setting for health-promoting choice architecture interventions because they reach the majority of working age population regularly. Published interventions have nevertheless been limited along several dimensions of scale-up, such as intervention settings, targets, strategies, and duration. Worksite choice architecture interventions for healthy lifestyles have mainly nudged food choices at worksite cafeterias [15, 16] or prompted stair use over the elevator [17] but rarely targeted eating or daily physical activity in other contexts at the workplace [18–20]. Equally rare are real-world interventions that have lasted longer than few months [21] or involved multiple implementation sites with broader target populations [22–25]. Furthermore, few choice architecture interventions have integrated implementation metrics in their effectiveness evaluations, albeit implementation influences the impact of health promotion programmes at workplaces [26] and other community settings [27].

Greater focus on implementation could assist the interpretation of study outcomes [28] and explain part of the heterogeneity observed in intervention effects. Within the choice architecture domain, effects may depend on, inter alia, the number [24] and type [8, 9] of intervention strategies implemented, the extent to which implementation covers behaviour-relevant contexts [24] and choice options [22] in the targeted environment, as well as the magnitude of modifications made to the choice architecture [23, 29, 30].

To advance understanding of the potential of the choice architecture approach to promote healthy lifestyles, we need wider-scale interventions and effectiveness evaluations that acknowledge implementation. We hence evaluated the effectiveness of a one-year quasi-experimental choice architecture intervention for healthy eating and daily physical activity. The evaluation was based on the dose (i.e., the number of intervention strategies applied) and quality of implementation. The intervention was conducted in real-world settings, adapted to local contexts, and integrated into the routine practices of diverse worksites. The study had two specific aims: (1) to assess intervention effectiveness on employees’ self-reported food consumption and physical activity patterns at work, and (2) to assess the association between implementation quality and employees’ self-reported perception of and response to the three most commonly applied intervention strategies.

## Methods

### Study design and setting

We rolled out a one-year hybrid type 2 implementation-effectiveness trial, StopDia at Work, between 2017 and 2019 in natural settings at workplaces from three regions of Finland (Northern Savo, South Karelia, and Päijät-Häme) [31]. The intervention aimed to promote healthy dietary choices and daily physical activity with subtle modifications to the worksite choice architecture. Hybrid type 2 designs have a dual focus on implementation and effectiveness outcomes, and they allow studying intervention effectiveness in new settings or populations while examining how to successfully implement the intervention [32]. Building on our implementation evaluation that was reported earlier [31], the current study evaluated the effectiveness of the StopDia at Work-intervention in a quasi-experimental pre-post design. The intervention was a part of a larger type 2 diabetes prevention study, Stop Diabetes (StopDia), that was approved by the research ethics committee of the hospital district of Northern Savo (statement 467/2016), Trial registration: NCT03156478 [33, 34].

### Participating worksites

Fifty-three distinct worksites participated in the intervention. The worksites represented sixteen medium-to-large organisations from various fields (industry, retail, education, municipality, farming, healthcare, and welfare), had physical work environments suitable for choice architectural modification, and employed altogether approximately 5100 employees. From the effectiveness evaluation, we excluded ten sites that represented two organisations: an institute of higher education (5 worksites, ∼ 370 employees) that moved to new premises halfway through the intervention and a retail operator (5 worksites, ∼ 360 employees) with incomplete data collection. From 25 worksites that represented three organisations, we received data only at the level of organisation instead of individual worksite. Hence, with these worksites, the organisations served as the observational units of analysis. Our final study sample comprised thus 21 observational units (representing 43 worksites, 14 organisations, and ∼ 4370 employees), which we refer to as “sites” (Additional file 1: Table S1). The sites represented both public (33%) and private (67%) sector and had a median of 46% (interquartile range, IQR 25–79%) male employees.

The management of participating sites gave their verbal informed consent for participation in the intervention. The employees of the intervention sites received general information on the larger Stop Diabetes study and the collaboration between their workplace and the study but were not disclosed the specific aim of the StopDia at Work-intervention to alter worksite choice architecture for healthy behaviours. This non-disclosure was to avoid interfering with employees’ natural perception of and response to the intervention.

Nineteen (90%) sites completed the full one-year intervention and two sites a slightly shorter 9-month intervention. The sites with the shorter duration were construction yards that completed their construction work after nine months, and the sites were closed.

### Intervention content and implementation

The content and implementation of the intervention were designed and contextualised to each participating worksite in collaboration between the research team and representatives of the worksites, as detailed earlier [31]. The representatives were local implementers selected among the personnel of the intervention sites. The implementers represented various occupational groups, including human resources (HR), occupational wellbeing, and work ability personnel; health and safety representatives; management; assistants; and catering staff. The co-design between the researchers and the implementers involved the selection of intervention strategies individually for each site from the StopDia Toolkit for creating health-promoting worksite environments. The toolkit was a hands-on instrument that described over 50 evidence-based strategies for modifying generic worksite choice architectures to facilitate healthy behaviours. The toolkit advanced the implementation of nutrition [35, 36] and physical activity [37, 38] guidelines and was informed by the nudge approach [1, 2], dual-systems models [4], and typologies of choice architecture interventions [39–41]. Additionally, the toolkit considered the needs and challenges of workplace health promotion that were identified in workshops and interviews conducted with contacted organisations over the recruitment process of the intervention [31].

The implementers of participating worksites delivered the intervention with the assistance of the research team. All adaptations maintained the essential elements of applied intervention strategies and were recorded carefully. In total 23 choice architecture strategies were employed across sites, sixteen for healthy eating and seven for daily physical activity (Table 1). The strategies modified the worksite choice architecture by altering the availability, position (visibility or proximity), functionality (convenience or default), presentation (attractiveness), size (tableware or portion), or information (primes, prompts, simplification, or references to social norms) of choice options, or by supporting self-regulation (commitment or reminders) required for the promoted behaviour. Strategies for healthy eating were typically implemented in coffee rooms, worksite cafeterias, or meetings, and strategies for daily physical activity in various common spaces, such as coffee rooms, copy rooms, monitoring rooms, bathrooms, or stairwells.

**Table 1:** Description of strategies implemented in the intervention

#	Strategy	Target/type (subtype)^1^	Setting
	HEALTHY EATING		
1.	Make healthy food/beverage options available.	Availability	Meetings
2.	Increase (decrease) the selection/variety of healthy (less healthy) options.	Availability	Cafeteria
3.	Replace less healthy options with nutritionally better alternatives.	Availability	Meetings
4.	Enhance the placement of healthy options.	Position (visibility, proximity)	Cafeteria
5.	Worsen the placement of less healthy options.	Position (visibility, proximity)	Cafeteria
6.	Serve fruit ready to eat.	Functionality (convenience)	Meetings
7.	Increase perceived variety by serving salad components from individual containers.	Position (visibility), Presentation (attractiveness)	Cafeteria
8.	Use smaller serving dishes for less healthy options.	Size (tableware)	Cafeteria
9.	Use smaller serving utensils for less healthy options.	Size (tableware)	Cafeteria
10.	Use smaller serving sizes for less healthy options.	Size (portion)	Meetings
11.	One plate-policy, i.e., no separate salad/bread plate at lunch.	Functionality (default), Size (tableware)	Cafeteria
12.	Facilitate the recognition of healthy options with the Heart Symbol-nutrition labels at the point of choice.	Information (simplification, prompt)	Cafeteria
13.	Cue better choices with “Follow the heart”-posters that facilitate the recognition of options labelled with the Heart Symbol-nutrition label.	Information (prime)	Cafeteria
14.	Facilitate and remind of drinking water by providing employees with personal, reusable water bottles.	Availability	Personal workstation
15.	Encourage smart packed lunches with a year-long recipe campaign featuring temptingly named and visually attractive packed lunch recipes. The recipes covered various types of packed lunch options, including warm courses, salads, smoothies, and sandwiches with season’s vegetables, fruit, and berries. The recipes met the nutritional criteria of national dietary guidelines but did not mention healthiness. Instead, they emphasised appealing sensory properties or ease of preparation. Campaign materials included one recipe for each week of the year, a poster, and a cardboard stand for printed recipe cards. The campaign slogan encouraged to form a habit of enjoying good packed lunches during breaks and featured a rhyme that encouraged to pick up a recipe card, stop by the store, and prepare, pack, and grab the packed lunch.	Presentation (attractiveness), Information (prompt, social norm)	Coffee rooms, lobbies, info screens, intranet, newsletters
16.	Encourage the provision of fruit at work by promoting and providing the “Fruit Crew”-starter set for forming fruit circles whose members take turns to organise fruit serving at work. The starter set included a poster that asked: “Already a member of the fruit crew?”, instructions and enrolment form, and a recyclable fruit basket.	Self-regulation (commitment, reciprocity), Information (prompt, social norm)	Coffee rooms
	DAILY PHYSICAL ACTIVITY		
17.	Enable active sitting with balance cushions or wobble stools.	Availability	Common spaces
18.	Encourage stair use with footprints leading to stairs.	Information (prompt), Self-regulation (reminder)	Stairwell
19.	Encourage stair use with the StopDia logo (a stop hand-sign with a heart on the palm) by the elevator.	Information (prompt), Self-regulation (reminder)	Elevator
20.	Encourage movement with posters depicting simple exercises suitable to be performed, e.g., by the copy machine, microwave, coffee maker, or bathroom.	Information (prompt)	Common spaces
21.	Make light exercise equipment available, e.g., gym sticks, balance boards, or hanging bars.	Availability	Common spaces
22.	Enhance the placement of exercise equipment.	Position (visibility, proximity)	Common spaces
23.	Encourage movement with a computer-based break exercise application.	Information (prompt), Self-regulation (reminder)	Personal workstation

Healthy foods were defined as compliant with the nutritional criteria of national dietary guidelines [36] and the Heart Symbol system of the Finnish Heart Association and the Finnish Diabetes association [42], which define product category-specific criteria for fat (quantity and quality), salt, sugar, and fibre

^1^Target or type of choice architectural modification with concepts compiled from existing frameworks of choice architecture interventions [9, 10, 39–41]

The median number of strategies implemented per site was four (range 2–14), a median of two (range 1–9) for healthy eating and one (range 1–5) for daily physical activity. The most common strategies were a packed lunch recipe campaign (#15) and a movement prompt strategy (#20) that all sites implemented, followed by a fruit crew-strategy (#16) that nine sites implemented (Table 1). These strategies could be delivered with print materials and/or digitally via info screens, emails, newsletters, or intranet. Participation in the intervention was free of charge for the sites, and the study provided materials for strategies that involved specific communication materials (#12, 13, 15, 16, 18, 19, 20). The sites were responsible for procuring any other materials needed for implementation, such as exercise equipment or new food products to worksite cafeterias.

### Data collection

The effectiveness evaluation used employee-level data collected with questionnaires pre and post intervention and site-level implementation data collected with implementer interviews and on-site observation halfway through and at the end of the intervention. The pre-intervention questionnaire was conducted immediately before intervention launch and the post intervention questionnaire a year later at the end of the intervention. At the two intervention sites that completed a shorter, 9-month intervention, the post intervention data collection took place at nine months. The sites launched the intervention in a schedule that was convenient for them between December 2017 and May 2018.

The employee questionnaires were designed to be brief to enable completion during a short break at work and to keep the threshold for completion low. The employees of intervention sites were invited to answer the questionnaires online via the Questback®-tool (www.questback.com) or with paper and pen, depending on which was feasible for the site. Site implementers forwarded the invitations and questionnaires from the research team to the employees. A cover letter informed that the questionnaire was anonymous, a part of the StopDia-study, and aimed to explore employees’ eating and physical activity habits at work. In the post intervention questionnaire, employees were encouraged to complete the questionnaire regardless of whether they had completed the pre intervention questionnaire. The collected questionnaire data comprised thus two cross-sectional datasets with partially overlapping samples. While the post intervention questionnaire enquired if the respondent had answered the pre intervention questionnaire as well, collected information did not enable linking individuals in the two datasets. Respondents gave their informed consent by voluntarily completing the questionnaire.

The site-level implementation data (implementer interviews and on-site observation) were collected over follow-up sessions at the intervention sites and/or via phone by the first two authors (ER, SV), as detailed elsewhere [31]. These authors were familiar with the intervention sites and the strategies the sites intended to implement. The authors had led the recruitment of participating organisations and the co-design of the intervention with the participating worksites. They also assisted the intervention sites in intervention implementation. The implementers who contributed to the data collection gave their verbal informed consent for participation.

### Measures

#### Employee characteristics and behavioural patterns at work

The questionnaires pre and post intervention collected information on the respondent’s predominant quality of work (physical vs. less physical), typical meal location (worksite cafeteria vs. else), and food consumption and physical activity patterns at work. The questionnaires asked the respondent to consider a typical work shift and respond accordingly. Data on the percentage of male employees per intervention site during the intervention year were received from site implementers.

Food consumption during a typical work shift was measured with six items that were adapted from a validated food frequency questionnaire (FFQ) [43] and selected as most relevant to the eating-related intervention strategies implemented. The items measured the consumption of vegetables and roots; fruit and berries; plain nuts, almonds, and seeds; sweet treats (e.g., confectionery, ice cream, chocolate, or sweets); fast food (e.g., meat pie, croissant, hamburger, sausage, or pizza); and water on a four-point scale (≥ 2 portions, 1 portion, < 1 portion, none). Additionally, we computed a diet quality score variable using the five FFQ-items of energy-containing foods (Additional file 1: Table S2). The score ranged from 0 to 26, a higher score reflecting higher diet quality at work. The scoring was based on a validated diet quality score, Healthy Diet Index (HDI) [44], that builds on the same FFQ as our questionnaires and evaluates adherence to a health-promoting diet congruent with the Nordic and Finnish nutrition recommendations.

Physical activity during a typical work shift was measured with three items, each with four response options, constructed to match the physical activity-related intervention strategies implemented. The items measured the performing of restorative movements (e.g., stretching), the use of exercise equipment when available (e.g., gym stick, therapy ball, hanging bar, or balance board), and the use of stairs when available. Regarding restorative movements and exercise equipment use, the response options were several times, once or twice, less than once, and never. Regarding stair use, the response options were always, frequently, seldom, and never. Respondents who reported never performing restorative movements or never using available exercise equipment were additionally asked about reasons for these choices.

#### Employees’ perception of and response to intervention

The post intervention questionnaire measured respondents’ perception of and response to the three most commonly applied intervention strategies: the packed lunch recipe campaign (#15, Table 1) and the movement prompt strategy (#20) that all sites implemented, and the fruit crew-strategy (#16) that nine sites implemented. The questionnaire asked the respondent to consider the past twelve months and facilitated responding with images of intervention materials. Regarding strategy #15, the questionnaire enquired whether the respondent had noticed the packed lunch recipes at their worksite, and if yes, whether the respondent had become interested in the recipes, and whether the respondent had tried the recipes. Regarding strategies #20 and #16, the questionnaire enquired whether the respondent had noticed corresponding intervention materials at the worksite, and if yes, whether they had acted upon them. The post intervention questionnaire also asked whether the respondent wished for support for healthy eating or physical activity from the employer, and whether the respondent had completed the pre intervention questionnaire.

#### Dose and quality of implementation at intervention sites

For a meaningful evaluation of intervention effectiveness on the measured food consumption and physical activity patterns, we organised the intervention strategies implemented at each site according to targeted behavioural patterns (Table 2). This categorisation enabled forming behaviour-specific implementation variables by multiplying the number of strategies implemented per behavioural pattern (i.e., dose) by their mean implementation quality (Additional file 1: Tables S3–S4). Implementation quality was evaluated by the first two authors (ER, SV) who independently rated each intervention strategy at each site at two follow-up time points (halfway through and at the end of the intervention) on a three-point scale (2 = successful, 1 = imperfect, 0 = failed) [31]. The evaluation built on an assessment framework that considered the essential elements of each strategy, fidelity to site-specific plans, the continuity of implementation, and accessibility to all employees. For behavioural patterns that were not targeted by specific strategies, i.e., the diet quality score and fast-food consumption (Table 2), we formed a global implementation variable of all eating-related intervention strategies implemented (Additional file 1: Table S3) to evaluate the effectiveness of the entire intervention.

**Table 2:** Strategies implemented to increase (↑) or decrease (↓) specific food consumption and physical activity patterns

Site	Strategies									
	↑ Vegetables/roots	↑ Fruit/berries	↑ Nuts/seeds	↓ Sweet treats	↓ Fast food	↑ Water	Other foods^2^	↑ Movement	↑ Exercise equipment	↑ Stairs
a. Kindergarten	15	15, 16	15	-	-	-	-	20	-	-
b. Factory	15	15	15	-	-	-	-	20	-	-
c. Grocery	15	15	15	-	-	-	-	20	-	-
d. Construction yard	15	15	15	-	-	-	-	20	-	-
e. Construction yard	15	15	15	-	-	-	-	20	-	-
f. Grocery	15	15	15	-	-	-	-	20	-	-
g. Construction yard	15	15	15	-	-	-	-	20	-	-
h. Construction yard	15	15	15	-	-	-	-	20	-	-
i. Social services centre	15	15, 16	15	-	-	-	-	20	-	-
j. Grocery	15	15, 16	15	-	-	-	-	20	-	-
k. Greenhouse	15	1, 15	15	10	-	-	-	20	-	-
l. Factory	15	15, 16	15	-	-	-	-	20, 21, 22	21, 22	-
m. Bureau	15	1, 15, 16	15	-	-	-	-	20, 23	-	-
n. Bureau	15	15	15	-	-	-	-	20	-	18,19
o. Office	15	1, 6, 15	15	3	-	-	-	20	-	-
p. Grocery	15	15, 16	15	-	-	-	-	20, 21, 22	21, 22	-
q. Bureau	15	15, 16	15	-	-	14	-	20, 21, 22	17, 21, 22	-
r. Bureau^1^	4, 12, 13, 15	1, 4, 15, 16	2, 4, 12, 13, 15	5	-	4, 12, 13	2, 4, 5, 12, 13	20, 21, 22	21, 22	-
s. Hospital^1^	7, 12, 13, 15	15, 16	2, 12, 13, 15	-	-	-	2, 4, 5, 12, 13	20	-	-
t. Factory^1^	4, 12, 13, 15	1, 2, 4, 12, 13, 15	2, 12, 13, 15	2, 10	-	4	2, 4, 5, 11, 12, 13	20	-	-
u. Factory^1^	2, 4, 12, 13, 15	1, 2, 4, 12, 13, 15	2, 4, 12, 13, 15	2, 5, 8	-	2, 4	1, 2, 4, 5, 9, 12, 13	20, 21, 22	21, 22	18, 19

Strategies: (1) enable healthy choices, (2) ↑/↓ selection, (3) replace with healthier alternatives, (4) ↑ visibility/proximity, (5) ↓ visibility/proximity, (6) ↑ convenience, (7) ↑ perceived variety, (8) ↓ serving dish size, (9) ↓ serving utensil size, (10) ↓ serving size, (11) one plate-policy, (12) prompt with point-of-choice Heart symbols, (13) prime with “Follow the heart”-posters, (14) provide personal water bottles, (15) promote packed lunch recipes, (16) promote the Fruit Crew-starter set, (17) enable active sitting, (18) prompt stair use with footprints, (19) prompt stair use with the StopDia logo, (20) prompt movement with posters, (21) ↑ exercise equipment availability, (22) ↑ exercise equipment visibility/proximity, (23) prompt movement with a break exercise application

^1^ Worksite cafeteria involved in the intervention

^2^ Strategies for other food consumption patterns, including dairy (milk, sour milk, yoghurt, cheese), whole grain (bread, sandwiches, porridge, snack biscuits, casseroles), fats (salad dressing, fat spread), meat (cold cuts, bacon), salted herring, olives, healthier pastries (sweet buns, berry pies), sugar-sweetened beverages, and lunch portion sizes (one plate-policy)

To control for the effect of strategies implemented that did not target but potentially influenced each behavioural pattern measured, we formed a complementary implementation variable for each behaviour-specific primary implementation variable. The complementary variables excluded the strategies that were used to form the corresponding primary implementation variables and included the remaining strategies related to food consumption (with food consumption patterns) or physical activity (with physical activity patterns). For example, if a site implemented strategies targeting fruit use, vegetable use, and sweet treat use, the behaviour-specific primary implementation variable of fruit use considered the strategies implemented for fruit use, whereas the complementary variable considered the remaining strategies that targeted vegetable and sweet treat use.

#### Outcomes

Primary outcomes were employees’ diet quality score; consumption of vegetables/roots, fruit/berries, nuts/almonds/seeds, sweet treats, fast food, and water; frequency of performing restorative movements, using exercise equipment, and using stairs during a typical work shift. Secondary outcomes were the noticing of, interest in, and trying of the packed lunch recipes (#15, Table 1); noticing of the fruit crew materials (#16) and joining a fruit crew; and noticing of and following the movement prompts (#20).

### Statistical analyses

Statistical analyses were performed with IBM SPSS statistics® version 29.0 (IBM Corp., Armonk, NY, USA), considering *p*-value 0.05 of a 2-tailed test an indication of statistical significance. We describe the analyses concisely here and provide more details in the supplementary material (Additional file 1).

### Intervention effectiveness on employees’ behavioural patterns at work

For the continuous diet quality score outcome, we fitted a linear mixed model with site-level random intercepts. For the categorical food consumption and physical activity outcomes, we fitted single-level multinomial logistic regression models because including site-level random intercepts resulted in model convergence issues. The convergence issues were often accompanied with estimates of negligible variation in the random intercepts, suggesting that ordinary single-level regression models would be an appropriate choice [45, 46]. Missing data ranged from 0.0 to 0.8% across the models.

The models included the main effect of time (post vs. pre intervention) and implementation (dose*quality), as well as their interaction, which was interpreted as intervention effectiveness. The interaction parameters describe how the log odds ratio of belonging to a certain outcome category post versus pre intervention changes depending on the level of implementation. We present these estimates at exponentiated scale, i.e., as ratios of two odds ratios (ORR). In multinomial models, the overall significance of the interaction was assessed with likelihood ratio test. We adjusted the models with relevant available site-level covariates: the proportion of male employees at the site during the intervention year, the proportion of respondents with physical work at each time point, and the proportion of respondents with a habit of eating at the worksite cafeteria at each time point (in models related to food consumption). These variables reflected the gender distribution, occupational status, and meal patterns of site employees—factors proven to influence diet and physical activity [47–51]. Models with the behaviour-specific implementation variables additionally included the complementary implementation variables and their interaction with time to adjust for the strategies implemented that did not target but potentially influenced the given behavioural outcome.

In multinomial models, we set the least beneficial outcome category as the reference level. With vegetables and roots; fruit and berries; nuts, almonds, and seeds; water; and all physical activity outcomes, the reference was the lowest category. With sweet treats and fast food, the reference was the highest consumption category. We used the original four-category outcome variables in all models except for the one related to water consumption, which was transformed into a three-category variable by merging the two lowest levels due to model identification issues. As a sensitivity analysis, we ran all the models also without the two sites with a shorter, 9-month intervention to control for the potential influence of premature termination.

### Association between implementation and employees’ perception of and response to intervention

We assessed the association between implementation quality and employees’ perception of and response to the three most commonly applied intervention strategies cross-sectionally based on post-intervention questionnaire data. For outcomes related to the packed lunch recipe campaign (#15, Table 1) and the movement prompt strategy (#20), we fitted mixed-effects logistic regression models with site-level random intercepts. For outcomes related to the fruit crew-strategy (#16), we used logistic regression models without site-level random intercepts due to convergence issues. Missing data ranged from 0.7 to 1.7% across the models.

The primary predictor of interest was the implementation quality of the outcome-related intervention strategy. Additionally, the models included relevant available site-level covariates: the proportion of male employees at the site during the intervention year, the proportion of respondents with physical work, the proportion of respondents who wished for support in healthy eating (in models related to #15–16) or physical activity (in models related to #20), and the proportion of respondents who reported having completed the questionnaire both pre and post intervention.

## Results

### Employee characteristics

The data collected among site employees comprised 1126 completed questionnaires pre intervention (median response rate across sites 34%, IQR 19–44%) and 943 completed questionnaires post intervention (median response rate 28%, IQR 23–58%) (Additional file 1: Table S1). The percentage of respondents with a physical work was 24% pre intervention and 23% post intervention. The percentage of respondents with a habit of eating at the worksite cafeteria was 23% at both time points. In the post intervention questionnaire, 24% reported that they had also completed the pre intervention questionnaire, 28% were not sure, and 46% had not.

### Dose and quality of implementation at intervention sites

Each intervention site implemented at least one strategy that encouraged the consumption of fruit and berries (range 1–6 strategies per site), vegetables and roots (range 1–5 strategies), and nuts, almonds, and seeds (range 1–5 strategies), and at least one strategy for the performing of restorative movements (range 1–3 strategies) (Table 2). Five sites (24%) targeted sweet treat consumption (range 1–3 strategies) and five sites exercise equipment use (range 2–3 strategies). Four sites (19%) implemented strategies for water consumption (range 1–3 strategies) and two sites (10%) for stair use (2 strategies each). Mean implementation quality (scale: 0–2) was overall high, with a site-level median of 1.8 (IQR 1.5–2) for all eating-related intervention strategies implemented and 1.7 (IQR 1.5–2) for all physical activity related strategies implemented (Additional file 1: Table S3–S4).

### Intervention effectiveness on employees’ behavioural patterns at work

#### Food consumption

Multinomial logistic regression models detected a statistically significant association between the intervention and a favourable change in employees’ fruit and berry consumption at work over the intervention year (interaction effect of time and implementation *p* = 0.006) (Table 3). The intervention was associated with an increase in the proportion of employees who consumed one portion (ORR 1.2, 95% CI 1.0 to 1.3) and the proportion who consumed two or more portions (ORR 1.2, 95% CI 1.0 to 1.4) of fruit and berries during a typical work shift compared to the proportion who consumed none. Additionally, the intervention had a significant association with an unfavourable change in employees’ sweet treat consumption (*p* = 0.048). The intervention was associated with a decrease in the proportion of employees who consumed less than one portion (ORR 0.6, 95% CI 0.4 to 1.0) and the proportion who consumed zero portions (ORR 0.6, 95% CI 0.4 to 0.9) of sweet treats during a typical work shift compared to the proportion who consumed at least two portions. No significant associations were observed between the intervention and changes in the diet quality score or in the consumption of vegetables and roots; nuts, almonds, and seeds; fast food; or water. Model results were robust to the exclusion of the two sites with a shorter intervention.

**Table 3:** Intervention effectiveness on employees’ behavioural patterns during a typical work shift

Outcome variable	n (%) pre^1^	n (%) post^1^	ORR (95% CI)^2^	*p*-value^3^
FOOD CONSUMPTION	1126	943		
Diet score (range 0–26 p.)	13 (9–17)	13.5 (9.5–18)	0.08 (-0.02; 0.18)	0.137
Vegetables/roots				0.849
≥ 2 portions	310 (27.5)	293 (31.1)	1.03 (0.69; 1.52)	
1 portion	432 (38.4)	376 (39.9)	1.09 (0.74; 1.58)	
< 1 portion	271 (24.1)	216 (22.9)	0.98 (0.66; 1.45)	
None	113 (10.0)	58 (6.2)	(ref)	
Fruit/berries				0.006
≥ 2 portions	216 (19.2)	184 (19.5)	1.22 (1.05; 1.41)	
1 portion	449 (39.9)	404 (42.8)	1.16 (1.01; 1.33)	
< 1 portion	283 (25.1)	254 (26.9)	1.03 (0.89; 1.19)	
None	178 (15.8)	101 (10.7)	(ref)	
Nuts/almonds/seeds				0.525
≥ 2 portions	36 (3.2)	29 (3.1)	0.98 (0.65; 1.47)	
1 portion	109 (9.7)	135 (14.3)	1.15 (0.93; 1.41)	
< 1 portion	344 (30.6)	325 (34.5)	1.08 (0.93; 1.24)	
None	637 (56.6)	454 (48.1)	(ref)	
Sweet treats				0.048
None	451 (40.1)	358 (38.0)	0.58 (0.35; 0.95)	
< 1 portion	546 (48.5)	473 (50.2)	0.60 (0.37; 0.99)	
1 portion	114 (10.1)	98 (10.4)	0.70 (0.42; 1.17)	
≥ 2 portions	15 (1.3)	14 (1.5)	(ref)	
Fast food				0.067
None	674 (59.9)	583 (61.8)	1.03 (0.88; 1.21)	
< 1 portion	347 (30.8)	288 (30.5)	1.08 (0.92; 1.27)	
1 portion	88 (7.8)	59 (6.3)	1.01 (0.85; 1.21)	
≥ 2 portions	17 (1.5)	13 (1.4)	(ref)	
Water				0.076
≥ 2 glasses	886 (78.7)	758 (80.4)	1.82 (1.03; 3.19)	
1 glass	168 (14.9)	137 (14.5)	1.70 (0.93; 3.11)	
< 1 glass or none	72 (6.4)	48 (5.1)	(ref)	
PHYSICAL ACTIVITY				
Performing of movements	1124	940		0.188
Several times	110 (9.8)	128 (13.6)	1.23 (0.99; 1.54)	
once or twice	396 (35.2)	330 (35.1)	1.15 (0.97; 1.37)	
Less than once	415 (36.9)	343 (36.5)	1.18 (1.00; 1.40)	
Never	203 (18.1)	139 (14.8)	(ref)	
Exercise equipment use^4^	386	405		0.040
Several times	9 (2.3)	15 (3.7)	1.78 (0.93; 3.40)	
once or twice	58 (15.0)	55 (13.6)	0.89 (0.70; 1.13)	
Less than once	109 (28.2)	105 (25.9)	0.82 (0.67; 1.00)	
Never	210 (54.4)	230 (56.8)	(ref)	
Stair use^4^	1030	881		0.170
Always	684 (66.4)	589 (66.9)	0.67 (0.33; 1.38)	
Frequently	227 (22.0)	212 (24.1)	0.76 (0.37; 1.57)	
Seldom	107 (10.4)	75 (8.5)	0.81 (0.39; 1.71)	
Never	12 (1.2)	5 (0.6)	(ref)	

^1^Frequencies (percentages) of valid observations pre and post intervention, except for the continuous diet score outcome, for which the data indicate medians (interquartile ranges)

^2^Exponentiated parameter estimates (95% confidence intervals) for the interaction of time and implementation

^3^Overall significance of the interaction effect of time and implementation in the model

^4^Among respondents who reported having exercise equipment/stairs available

#### Daily physical activity

Multinomial logistic regression models detected a statistically significant association between the intervention and changes in the frequency at which employees used available exercise equipment at work (interaction effect of time and implementation *p* = 0.040) (Table 3). Estimates suggested the intervention was associated with a decrease in the proportion of employees who used the equipment up to two times per work shift and with an increase in the proportion who used the equipment several times per work shift compared to the proportion who never used the equipment. No significant associations were observed between the intervention and changes in the performing of restorative movements or stair use. Model results were robust to the exclusion of the two sites with a shorter intervention.

Reasons for never performing restorative movements or never using available exercise equipment were abundant (Additional file 1: Table S5). The most common reasons across time points were that the idea never crossed one’s mind; forgetting; the lack of time, space, or motivation; and embarrassment.

#### Association between implementation and employees’ perception of and response to intervention

In the post intervention questionnaire, most respondents reported that they had noticed the packed lunch recipes (70%), the fruit crew-materials (84%), and the movement prompts (76%) (Table 4). Of these respondents, respectively, 67% had become interested in and 31% had tried at least one recipe, 28% had joined a fruit crew, and 50% had followed the movement prompts. In the post intervention sample, the proportion of respondents who wished that the employer would provide support for healthy eating was 37%, and the proportion who wished for support for physical activity was 61%.

**Table 4:** Association between implementation quality and employees’ perception of and response to three specific intervention strategies

Outcome variable	n (%)	OR (95% CI)	*p*-value
Packed lunch recipes			
Noticed materials	932		
Yes	649 (69.6)	5.42 (1.05; 27.83)	0.044
No	283 (30.4)	(ref.)	
Became interested in at least one recipe^1^	645		
Yes	434 (67.3)	1.19 (0.65; 2.20)	0.565
No	211 (32.7)	(ref.)	
Tried at least one recipe^1^	646		
Yes	203 (31.4)	2.32 (1.19; 4.54)	0.017
No	443 (68.6)	(ref.)	
Fruit crew-starter set^2^			
Noticed materials	533		
Yes	448 (84.1)	0.40 (0.20; 0.84)	0.015
No	85 (15.9)	(ref.)	
Joined a fruit crew^1^	444		
Yes	122 (27.5)	2.94 (1.82; 4.73)	< 0.001
No	322 (72.5)	(ref.)	
Movement prompts			
Noticed materials	928		
Yes	701 (75.5)	5.28 (0.86; 32.37)	0.067
No	227 (24.5)	(ref.)	
Followed the prompts^1^	701		
Yes	351 (50.1)	1.14 (0.57; 2.24)	0.633
No	350 (49.9)	(ref.)	

^1^Among respondents who noticed the materials

^2^Among respondents (*n* = 537) of the nine sites that implemented the fruit crew-strategy

Logistic regression models indicated that the quality of implementation was positively associated with the odds of noticing (OR 5.4, 95% CI 1.1 to 27.8) and trying (OR 2.3, 95% CI 1.2 to 4.5) the packed lunch recipes but unrelated with the odds of becoming interested in the recipes (OR 1.2, 95% CI 0.6 to 2.2) (Table 4). With the fruit crew-strategy, the quality of implementation was negatively associated with the odds of noticing the fruit crew materials (OR 0.4, 95% CI 0.2 to 0.8) yet positively associated with the odds of joining a fruit crew (OR 2.9, 95% CI 1.8 to 4.7). Implementation quality was not significantly associated with the odds of noticing or following the movement prompts.

## Discussion

This study evaluated the effectiveness of a contextualised, multicomponent choice architecture intervention for healthy eating and daily physical activity conducted in real-world settings at heterogeneous worksites. Building on the interaction effect of time and site-specific dose and quality of implementation, the evaluation found the intervention significantly associated with a favourable change in employees’ fruit and berry consumption and with an unfavourable change in sweet treat consumption at work over the one-year intervention. The intervention was also significantly associated with a change in the use of exercise equipment, but the meaning of this association was less straightforward to interpret. Associations with changes in other behavioural outcomes were non-significant. Implementation quality was positively associated with the perception of and response to the packed lunch recipes, and with response to the fruit crew-strategy.

### Intervention effectiveness on employees’ behavioural patterns at work

#### Food consumption

The strongest evidence we found on the effectiveness of the intervention concerned the consumption of fruit and berries. The intervention was associated with increased fruit and berry consumption, and the strength of this association seemed to increase consistently from the lowest to the highest consumption level. Intervention sites implemented up to six strategies for fruit and berry consumption. An increased number of strategies meant greater diversity in the types of strategies used and in the mechanisms through which the strategies supposedly influence behaviour. Noteworthy, sites with greater dose and quality of implementation applied not only cognitively or affectively oriented strategies that influenced behaviour via reflective processes (i.e., the packed lunch recipes, the fruit crew-starter set, visibility enhancements, and/or nutrition labels) but also behaviourally oriented strategies that tangibly reduced the physical effort required to choose and consume fruit at work (i.e., increased availability and/or convenience). At sites with greater dose and quality of implementation, the intervention also targeted several eating-related contexts at the worksite (coffee rooms, meetings, and/or cafeterias). Consistent with our findings, other worksite choice architecture interventions have observed favourable effects on food consumption after implementing various types of strategies that function through various mechanisms (availability, visibility, proximity, promotion, and price incentives) [24] and after reducing effort with enhanced relative availability [23] and/or convenience [19] of targeted foods. Meta-analyses also suggest that behaviourally oriented strategies in general yield on average greater effects compared to cognitively or affectively oriented strategies [8, 9]. A further factor that may explain the association the present study found between the intervention and a favourable change in fruit and berry consumption is that fruit are a practical snack at work.

Besides fruit and berries, we detected no favourable associations between the intervention and changes in the consumption of other foods. For foods other than fruit and berries, sites used mainly subtle cognitively or affectively oriented strategies that demanded greater deliberation, motivation, and agency from the employees. While our acceptability evaluation that was based on implementer interviews and an employee questionnaire indicated that the strategies employed in the intervention were overall well received [52], the strategies were unlikely able to appeal to each individual in the broad target population, thus reducing effectiveness [8]. This rationale receives support from our field experiment at a worksite cafeteria that found three cognitively oriented strategies—priming health messages, prominent nutrition labels, and minor visibility enhancements—ineffective in improving food choices among customers who prioritised sensory appeal and familiarity [53]. On the contrary, health messages and labels accompanied with improved availability and/or visibility proved effective in a hospital cafeteria [21, 54, 55] and in a military dining hall [56]—contexts where health and fitness were likely appreciated.

Unexpectedly, the intervention appeared associated with an unfavourable change in sweet treat consumption. This association has at least two possible explanations. First, the strategies that reduced the serving sizes of sweet treats or replaced available sweet treat options with nutritionally better alternatives may have increased the number of portions consumed. Second, observations from intervention sites revealed that the reductions made to the visibility, proximity, or availability of sweet treats were overall small and covered only a part of the contexts at the worksites that provided sweet temptations and only a part of the sweet treat options available in these contexts. Prior research has found relatively small changes to visibility and availability ineffective in reducing the sales of snacks, such as candy and confectionery at worksite cafeterias [24]. Reviews on proximity [29, 30] strategies also suggest that intervention effects are proportionate to the magnitude of modifications. At the same time, reducing sweet treat consumption may be more challenging than increasing healthy food consumption and might thus require substantial changes to the physical and social worksite environment. The availability of indulging foods that conflict with attempts to eat healthily challenges self-regulation [57] and can trigger deliberate reasoning processes that justify the indulgence—as portrayed by the self-licensing effect [58, 59]. Providing sweet treats and enjoying them with colleagues can also be an important part of the work culture, with social norms preventing refusals [57].

#### Daily physical activity

The intervention appeared associated with a reduction in the proportion of employees with infrequent use of available exercise equipment yet an increase in the proportion with frequent use of the equipment, as compared with the proportion who never used the equipment. The meaning of these findings remains unclear, however, as the data do not support a straightforward interpretation. No significant associations were observed with other physical activity outcomes. While a meta-analysis suggested eating behaviour to be particularly responsive to choice architecture interventions [8], increasing daily physical activity may require stronger guidance and support from the social and organisational environment. The proportion of our questionnaire respondents who wished for support in physical activity from the employer was markedly greater than the proportion of respondents who wished for support in healthy eating. Common reasons for never performing restorative movements or using exercise equipment at work included forgetting, lack of time or space, and embarrassment. The importance of a supportive social environment was demonstrated in an intervention for increased walking at the workplace [20]. In this intervention, a digital app that promoted social support and social comparison through team challenges was effective in increasing employees’ daily step count, but motivational messages and point-of-choice prompts in the worksite choice architecture failed to maintain the achieved effects [20].

#### Association between implementation and employees’ perception of and response to intervention

Based on the self-reported perception of intervention materials, the three most commonly applied intervention strategies (i.e., the packed lunch recipes, the fruit crew-strategy, and movement prompts) reached a strong majority of respondents. This finding reflects the overall high implementation quality across intervention sites and supports earlier evidence according to which prominently displayed intervention materials capture visual attention [53].

Higher quality of implementation predicted the noticing and trying of the packed lunch recipes but was unrelated to becoming interested in the recipes. This suggests that the effect of the quality of implementation on behaviour be mediated predominantly via noticing. Once the recipes were noticed, implementation had little influence on whether employees became interested in them. The finding is logical considering the strong and stable food preferences people often have. Emerging evidence suggests people are more likely to act upon choice architecture interventions when they agree with or hold no strong preferences against the nudged behaviours; thus validating the legitimacy of choice architecture interventions [60]. As supposed by the core principles of the choice architecture approach [2], interventions seem to maintain people’s freedom to choose according to their preferences.

Interestingly, we observed higher implementation quality to decrease the odds of noticing the fruit crew materials yet increase the odds of joining a fruit crew. This counter-intuitive finding could be explained by the overall high rate of noticing the materials and by our implementation quality assessment that omitted intervention launch. At the sites with the lowest quality ratings, the fruit crew-materials were delivered successfully at the launch of the intervention but by the first follow-up assessment halfway through the intervention, the implementation had ceased. Nevertheless, all the respondents from these sites reported that they had noticed the materials. The successful launch thus likely facilitated the noticing of materials, while the soon fading implementation discouraged seizing on them. Another possible explanation is that at sites with successful implementation, the focus was on the activity of forming fruit crews and organising fruit serving at the worksite with less attention paid on the provided intervention materials.

### Strengths and limitations

The strengths of this study include a theory- and evidence-based intervention conducted in real-world settings at over twenty diverse worksites by integrating the intervention into the routine operations of the sites. For enhanced feasibility and acceptability, the intervention was designed and contextualised to each participating worksite in collaboration with the sites. The sites applied a broad range of choice architecture strategies whose implementation was monitored systematically at two follow-up time points. The work produced thus evidence on over twenty unique implementations. Building on a mixed-methods evaluation of implementation [31] and employee-level self-reports pre and post intervention, the study developed an approach to evaluate effectiveness by considering the dose and quality of implementation relevant to each outcome measured. The study contributes to the translation and upscaling of choice architecture interventions from more controlled research settings to diverse real-world operations, providing insights on the effectiveness of the choice architecture approach in the workplace context.

Key limitations of the study include the lack of control group, scarce information available on the employees who completed the questionnaires, partly overlapping samples with no possibility to link individuals in the pre and post intervention datasets, a relatively low questionnaire response rate at the participating worksites, and reliance on error-prone self-reported data on employees’ perception and behaviour. These limitations increase uncertainty in the study outcomes. Whilst we had no proper control group, we had intervention sites with varying levels of implementation. This enabled us to consider the intervention as a continuous variable and assess the effectiveness of incremental increases in the dose and quality of implementation. With half of the primary outcomes, the smallest number of outcome-related strategies implemented per site was zero. With the other half, the smallest number was one. While the data did not enable assessing the effectiveness of individual intervention strategies, this was not the purpose of the study in the first place. Prior research has produced evidence on the efficacy of individual choice architecture strategies. The current intervention focused thus on their wider-scale implementation in real-world circumstances. The intervention was designed to increase our understanding of the overall feasibility [31], acceptability [52], and effectiveness of the choice architecture approach in the workplace context.

Without identifiable data on questionnaire respondents, we were unable to track individuals from baseline to follow up, to evaluate the extent to which the respondents represented the personnel of the participating worksites, or to examine the effects of individual characteristics on intervention effectiveness. Yet, we adjusted statistical analyses with relevant available site-level covariates, including the proportion of male employees, respondents with physical work, respondents eating at the worksite cafeteria, and respondents who completed the questionnaire both pre and post intervention. The decision to limit data collection to unidentifiable data was related to our choice not to disclose to site employees the specific aim of the intervention, which was to modify worksite choice architecture for healthy behaviours. At the time, it was unclear whether such disclosure would influence employees’ perception of and response to the intervention. Later on, research has touched upon the topic and suggests that study subjects’ awareness of the presence, purpose, or working mechanism of choice architecture interventions does not reduce intervention effectiveness [60]. Future studies could hence inform their target populations more freely of implemented interventions.

The food consumption and physical activity patterns measured in this study covered time spent at work and were hence unable to reveal changes in behavioural patterns outside working hours. Covered food consumption patterns were limited to six key food groups most relevant to the intervention strategies implemented, and the FFQ-items used to measure food consumption were quite crude. Thus, the available data provides merely suggestive evidence on the effectiveness of the intervention on the consumption frequency of diverse food types. The rationale for the brief data collection was the aim to design a questionnaire that could be completed with minimal effort during a short break at work. This methodological choice was assumed to result in greater response rates.

The constructed implementation variables had their limitations as well. Implementation dose, measured as the number of intervention strategies applied, did not consider the type of intervention strategy or the mechanism through which it was expected to change behaviour, although these characteristics have proved to influence effect sizes [8, 9]. Implementation quality, in turn, was measured on a three-point scale that was rather insensitive to variations in diverse aspects of implementation, such as the extent to which implementation covered relevant contexts and available choice options in the worksite environment, and the magnitude of modifications made to the targeted choice architecture. Additionally, the quality assessment was based on merely two follow-up measurements over the one-year intervention.

### Implications for practice and research

For more effective future interventions, we recommend workplaces to employ intervention strategies that reduce the physical effort required from employees to eat well and stay active at work, and that cover all relevant behavioural contexts and available choice options at the worksite. Relying on strategies that encourage desired choices with enhanced visibility or subtle visual or written cues may not be enough, particularly if not tailored to the target group’s behavioural goals and preferences. For increased physical activity, efforts to build a supportive social and organisational environment may also be required. For more accurate estimates of the effectiveness of choice architecture interventions in the real world, future studies should adopt stronger study designs and invest in the quality and quantity of data collected on intervention implementation and the target audience’s characteristics and behaviour.

## Conclusions

This study evaluated the effectiveness of a contextualised, multicomponent, and year-long choice architecture intervention for healthy eating and daily physical activity conducted in real-world settings at heterogeneous worksites. The evaluation built on the interaction effect of time and site-specific dose and quality of implementation. Results suggested that the intervention had a positive influence on employees’ fruit and berry consumption at work. Likely contributing to this finding, sites with greater dose and quality of implementation targeted fruit and berry consumption by employing intervention strategies that tangibly reduced the physical effort required to choose and consume fruit or berries at work and by extending intervention delivery to multiple eating-related contexts at the worksite. Moreover, results suggested that higher implementation quality can positively influence the perception of and response to cognitively or affectively oriented choice architecture strategies. This finding, however, varied along the strategy implemented.

### Electronic supplementary material

Below is the link to the electronic supplementary material.

Supplementary Material 1

## Data Availability

The datasets used and/or analysed during the current study are available from the corresponding author on a reasonable request.
