# Supplementary material for: Effectiveness of workplace choice architecture modification for healthy eating and daily physical activity
Source: BMC Public Health. 2024 Apr 1;24:939. doi: 10.1186/s12889-024-18482-1 (PMC10986070; doi:10.1186/s12889-024-18482-1)
Supplement: Supplementary file 1 — Supplementary Material 1 [file 12889_2024_18482_MOESM1_ESM.pdf]

Supplementary material

Effectiveness of workplace choice architecture modification for healthy eating and daily physical activity

Content

Participating worksites 1
Table S1. Characteristics of intervention sites and questionnaire data collected pre and post intervention. 1
Measures..2
Table S2. Formation of the diet score variable based on the Healthy Diet Index (Lindström et al., 2021)..............2
Table S3. Formation and descriptive statistics of implementation variables related to eating 3
Table S4. Formation and descriptive statistics of implementation variables related to physical activity.................4
Statistical analyses..5
Results 5
Table S5. Reasons for never performing restorative movements or using available exercise equipment. 5
References 6

Participating worksites

Table S1. Characteristics of intervention sites and questionnaire data collected pre and post intervention.

Site	Organisation/field	Sector	% Men	Pre<br>n (%)	Physical	Café	Post<br>n (%)	Physical	Café
a. Kindergarten	Municipality	Public	7	13 (43)	62	15	9 (30)	44	22
b. Factory	Food industry	Private	70	59 (24)	22	24	71 (28)	31	14
c. Grocery	Retail	Private	18	11 (15)	73	0	20 (27)	55	0
d. Construction yard	Construction industry	Private	100	12 (28)	92	0	21 (49)	81	0
e. Construction yard	Construction industry	Private	97	8 (25)	100	0	20 (63)	55	0
f. Grocery (3 worksites combined)	Retail	Private	20	102 (34)	45	2	42 (14)	60	5
g. Construction yard	Construction industry	Private	100	19 (63)	68	0	8 (27)	63	0
h. Construction yard	Construction industry	Private	100	14 (54)	43	0	9 (35)	56	0
i. Social services centre	Welfare	Public	5	17 (43)	0	0	27 (68)	0	0
j. Grocery	Retail	Private	18	29 (17)	52	0	39 (23)	62	3
k. Greenhouse	Farming	Private	35	24 (17)	46	0	12 (9)	33	0
l. Factory	Metal industry	Private	80	85 (14)	18	14	33 (6)	3	21
m. Bureau (2 worksites combined)	Municipality	Public	45	100 (45)	3	29	135 (61)	1	35
n. Bureau	Municipality	Public	29	25 (36)	4	52	47 (67)	4	62
o. Office	Construction industry	Private	56	22 (44)	5	0	21 (42)	0	0
p. Grocery	Retail	Private	18	46 (61)	57	0	49 (65)	61	0
q. Bureau	Municipality	Public	39	40 (50)	3	5	46 (58)	2	4
r. Bureau[1]	Municipality	Public	20	74 (25)	5	22	70 (23)	4	26
s. Hospital (20 worksites combined)[1]	Healthcare	Public	46	91 (19)	24	40	129 (26)	22	41
t. Factory[1]	Chemical industry	Private	75	152 (38)	24	48	8 (2)	63	25
u. Factory[1]	Forest industry	Private	78	183 (19)	13	34	127 (13)	16	36

%Men: percentage of male employees at the site during the intervention year
n (%): number of completed questionnaires (response rate)
Physical: percentage of respondents with a physical job
Café: percentage of respondents who typically ate at the worksite cafeteria
[1]Worksite cafeteria involved in the intervention.

Measures

Table S2. Formation of the diet score variable based on the Healthy Diet Index (Lindström et al., 2021)

Healthy Diet Index Portions	Score	StopDia at Work Portions per work shift	Score	Maximum	% Maximum
Vegetables and roots[1]					
≥ 3/day	12				
2/day	8	≥ 2	10*	10	38
1/day	4	1	4		
4-6/week	2				
1-3/week	1	< 1	1.5*		
< 1/week or none	0	none	0		
Fruit and berries[2]					
≥ 2/day	8	≥ 2	8	8	31
1/day	5	1	5		
4-6/week	2				
1-3/week	1	< 1	1.5*		
< 1/week or none	0	none	0		
Nuts, almonds, and seeds[3]					
≥ 2/day	2	≥ 2	2	2	8
1/day	2	1	2		
4-6/week	1				
1-3/week	1	< 1	1*		
< 1/week or none	0	none	0		
Sweet treats[4]					
≥ 2/day	0	≥ 2	0		
1/day	0	1	0		
4-6/week	1				
1-3/week	2	< 1	1.5*		
< 1/week or none	3	none	3	3	12
Fast food[5]					
≥ 1/day	0	≥ 2	0		
		1	0		
4-6/week	0				
1-3/week	1				
1-3/month	2	< 1	1*		
< 1/month or none	3	none	3	3	12
Maximum				26	100

[1]portion = e.g., a medium-sized carrot or 1 dl of salad or grated or cooked vegetables
[2]portion = a medium-sized fruit or 1 dl of berries
[3]portion = 2 tablespoons or 30 g
[4]portion = e.g., a piece of pie or cake, a Danish pastry or doughnut, 3–4 cookies, an ice cream cornet, or a chocolate bar
[5]portion = e.g., a meat pie, a hamburger, or a slice of pizza
*Scoring equals the average of corresponding servings in the Healthy Diet Index.

Table S3. Formation and descriptive statistics of implementation variables related to eating. D = dose, Q = mean quality (range 0–2), # = intervention strategy

	Vegetable/root			Fruit/berry			Nut/almond/seed			Sweet treat			Fast food			Water			Global		
	D (#)	Q	D*Q	D (#)	Q	D*Q	D (#)	Q	D*Q	D (#)	Q	D*Q	D (#)	Q	D*Q	D (#)	Q	D*Q	D (#)	Q	D*Q
Site																					
a. Kindergarten	1 (#15)	1	1	2 (#15, 16)	0.8	1.5	1 (#15)	1	1	0	0	0	0	0	0	0	0	0	2 (#15, 16)	0.8	1.5
b. Factory	1 (#15)	2	2	1 (#15)	2	2	1 (#15)	2	2	0	0	0	0	0	0	0	0	0	1 (#15)	2	2
c. Grocery	1 (#15)	1.5	1.5	1 (#15)	1.5	1.5	1 (#15)	1.5	1.5	0	0	0	0	0	0	0	0	0	1 (#15)	1.5	1.5
d. Construction yard	1 (#15)	2	2	1 (#15)	2	2	1 (#15)	2	2	0	0	0	0	0	0	0	0	0	1 (#15)	2	2
e. Construction yard	1 (#15)	2	2	1 (#15)	2	2	1 (#15)	2	2	0	0	0	0	0	0	0	0	0	1 (#15)	2	2
f. Grocery[1]	1 (#15)	2	2	1 (#15)	2	2	1 (#15)	2	2	0	0	0	0	0	0	0	0	0	1 (#15)	2	2
g. Construction yard	1 (#15)	2	2	1 (#15)	2	2	1 (#15)	2	2	0	0	0	0	0	0	0	0	0	1 (#15)	2	2
h. Construction yard	1 (#15)	2	2	1 (#15)	2	2	1 (#15)	2	2	0	0	0	0	0	0	0	0	0	1 (#15)	2	2
i. Social services centre	1 (#15)	2	2	2 (#15, 16)	1	2	1 (#15)	2	2	0	0	0	0	0	0	0	0	0	2 (#15, 16)	1	2
j. Grocery	1 (#15)	1	1	2 (#15, 16)	1.5	3	1 (#15)	1	1	0	0	0	0	0	0	0	0	0	2 (#15, 16)	1.5	3
k. Greenhouse	1 (#15)	2	2	2 (#1, 15)	2	4	1 (#15)	2	2	1 (#10)	2	2	0	0	0	0	0	0	3 (#1, 10, 15)	2	6
l. Factory	1 (#15)	1	1	2 (#15, 16)	1	2	1 (#15)	1	1	0	0	0	0	0	0	0	0	0	2 (#15, 16)	1	2
m. Bureau[1]	1 (#15)	2	2	3 (#1, 15, 16)	1.5	4.5	1 (#15)	2	2	0	0	0	0	0	0	0	0	0	3 (#1, 15, 16)	1.5	4.5
n. Bureau	1 (#15)	2	2	1 (#15)	2	2	1 (#15)	2	2	0	0	0	0	0	0	0	0	0	1 (#15)	2	2
o. Office	1 (#15)	2	2	3 (#1, 6, 15)	1.7	5	1 (#15)	2	2	1 (#3)	2	2	0	0	0	0	0	0	4 (#1, 3, 6, 15)	1.8	7
p. Grocery	1 (#15)	1	1	2 (#15, 16)	1.5	3	1 (#15)	1	1	0	0	0	0	0	0	0	0	0	2 (#15, 16)	1.5	3
q. Bureau	1 (#15)	2	2	2 (#15, 16)	2	4	1 (#15)	2	2	0	0	0	0	0	0	1 (#14)	2	2	3 (#14, 15, 16)	2	6
r. Bureau[2]	4 (#4, 12, 13, 15)	1.1	4.5	4 (#1, 4, 15, 16)	1.5	6	5 (#2, 4, 12, 13, 15)	1.1	5.5	1 (#5)	1	1	0	0	0	3 (#4, 12, 13)	0.8	2.5	8 (#1, 2, 4, 5, 12, 13, 15, 16)	1.2	9.5
s. Hospital[1, 2]	4 (#7, 12, 13, 15)	1.7	6.6	2 (#15, 16)	1.1	2.2	4 (#2, 12, 13, 15)	1.9	7.6	0	0	0	0	0	0	0	0	0	8 (#2, 4, 5, 7, 12, 13, 15, 16)	1.7	13.2
t. Factory[2]	4 (#4, 12, 13, 15)	1.8	7	6 (#1, 2, 4, 12, 13, 15)	1.8	11	4 (#2, 12, 13, 15)	1.8	7	2 (#2, 10)	2	4	0	0	0	1 (#4)	2	2	9 (#1, 2, 4, 5, 10, 11, 12, 13, 15)	1. 9	17
u. Factory[2]	5 (#2, 4, 12, 13, 15)	1.1	5.5	6 (#1, 2, 4, 12, 13, 15)	1.3	7.5	5 (#2, 4, 12, 13, 15)	1.1	5.5	3 (#2, 5, 8)	1.5	4.5	0	0	0	2 (#2, 4)	1.3	2.5	9 (#1, 2, 4, 5, 8, 9, 12, 13, 15)	1.4	13
Descriptive statistics																					
Min	1	1	1	1	0.8	1.5	1	1	1	0	0	0	0	0	0	0	0	0	1	0.8	1.5
Q1	1	1.1	2	1	1.5	2	1	1.1	2	0	0	0	0	0	0	0	0	0	1	1.5	2
Median	1	2	2	2	1.7	2	1	2	2	0	0	0	0	0	0	0	0	0	2	1.8	2
Q4	1	2	2	2	2.0	4	1	2	2	0	0	0	0	0	0	0	0	0	3	2	6
Max	5	2	7	6	2.0	11	5	2	7.6	3	2	4.5	0	0	0	3	2	2.5	9	2	17

Intervention strategies (#): 1. enable healthy choices, 2. ↑/↓ selection, 3. replace with healthier alternatives, 4. ↑ visibility/proximity, 5. ↓ visibility/proximity, 6. ↑ convenience, 7. ↑ perceived variety, 8. ↓ serving dish size, 9. ↓ serving utensil size, 10. ↓ serving size, 11. one plate-policy, 12. prompt with point-of-choice Heart symbols, 13. prime with "Follow the heart"-posters, 14. provide personal water bottles, 15. promote packed lunch recipes, 16. promote the Fruit Crew-starter set.

The table shows the mean implementation quality ratings of corresponding intervention strategies with values rounded to one decimal place. The mean values represent the average of individual strategies whose quality was rated at two time points, halfway through and at the end of the intervention.

[1] Data of 3 (grocery), 2 (bureau), and 20 (hospital) worksites combined.

[2] Worksite cafeteria involved in the intervention.

Table S4. Formation and descriptive statistics of implementation variables related to physical activity. D = dose, Q = mean quality (range 0–2), # = intervention strategy

		Movement			Exercise equipment			Stair use			Global		
		D (#)	Q	D*Q	D (#)	Q	D*Q	D (#)	Q	D*Q	D (#)	Q	D*Q
Site													
a.	Kindergarten	1 (#20)	1	1	0	0	0	0	0	0	1 (#20)	1	1
b.	Factory	1 (#20)	1	1	0	0	0	0	0	0	1 (#20)	1	1
c.	Grocery	1 (#20)	2	2	0	0	0	0	0	0	1 (#20)	2	2
d.	Construction yard	1 (#20)	1.5	1.5	0	0	0	0	0	0	1 (#20)	1.5	1.5
e.	Construction yard	1 (#20)	1.5	1.5	0	0	0	0	0	0	1 (#20)	1.5	1.5
f.	Grocery[1]	1 (#20)	2	2	0	0	0	0	0	0	1 (#20)	2	2
g.	Construction yard	1 (#20)	2	2	0	0	0	0	0	0	1 (#20)	2	2
h.	Construction yard	1 (#20)	2	2	0	0	0	0	0	0	1 (#20)	2	2
i.	Social services centre	1 (#20)	2	2	0	0	0	0	0	0	1 (#20)	2	2
j.	Grocery	1 (#20)	2	2	0	0	0	0	0	0	1 (#20)	2	2
k.	Greenhouse	1 (#20)	1	1	0	0	0	0	0	0	1 (#20)	1	1
l.	Factory	3 (#20, 21, 22)	1.7	5	2 (#21, 22)	2	4	0	0	0	3 (#20, 21, 22)	1.7	5
m.	Bureau[1]	2 (#20, 23)	1.6	3.3	0	0	0	0	0	0	2 (#20, 23)	1.6	3.3
n.	Bureau	1 (#20)	2	2	0	0	0	2 (#18,19)	2	4	3 (#18, 19, 20)	2	6
o.	Office	1 (#20)	1	1	0	0	0	0	0	0	1 (#20)	1	1
p.	Grocery	3 (#20, 21, 22)	2	6	2 (#21, 22)	2	4	0	0	0	3 (#20, 21, 22)	2	6
q.	Bureau	3 (#20, 21, 22)	1.7	5	3 (#17, 21, 22)	1.3	4	0	0	0	4 (#17, 20, 21, 22)	1.5	6
r.	Bureau[2]	3 (#20, 21, 22)	1.7	5	2 (#21, 22)	1.5	3	0	0	0	3 (#20, 21, 22)	1.7	5
s.	Hospital[1, 2]	1 (#20)	1.5	1.5	0	0	0	0	0	0	1 (#20)	1.5	1,5
t.	Factory[2]	1 (#20)	2	2	0	0	0	0	0	0	1 (#20)	2	2
u.	Factory[2]	3 (#20, 21, 22)	2	6	2 (#21, 22)	2	4	2 (#18,19)	2	4	5 (#18, 19, 20, 21, 22)	2	10
Descriptive statistics													
	Min	1	1	1	0	0	0	0	0	0	1	1	1
	Q1	1	1.5	1.5	0	0	0	0	0	0	1	1.5	1.5
	Median	1	1.7	2	0	0	0	0	0	0	1	1.7	2
	Q4	2	2	3.3	0	0	0	0	0	0	3	2	5
	Max	3	2	6	3	2	4	2	2	4	5	2	10

Intervention strategies (#): 17. enable active sitting, 18. prompt stair use with footprints, 19. prompt stair use with the StopDia logo, 20. prompt movement with posters, 21. ↑ exercise equipment availability, 22. ↑ exercise equipment visibility/proximity, 23. prompt movement with a break exercise application.

The table shows the mean implementation quality ratings of corresponding intervention strategies with values rounded to one decimal place. The mean values represent the average of individual strategies whose quality was rated at two time points, halfway through and at the end of the intervention.

[1] Data of 3 (grocery), 2 (bureau), and 20 (hospital) worksites combined.

[2] Worksite cafeteria involved in the intervention.

Statistical analyses

We studied the effectiveness of the StopDia at Work-intervention on the defined outcomes with mixed-effects models and conventional regression models. Mixed-effects models were specified with a 2-level data structure using site (n=21) as the clustering variable. We built linear mixed models for continuous outcomes and generalised linear mixed models for categorical outcomes, respectively, with the MIXED and GENLINMIXED routines of IBM SPSS statistics® version 29.0 (IBM Corp., Armonk, NY, USA). The default estimation method SPSS employs is restricted maximum likelihood (REML) in MIXED (Heck et al., 2021, p. 20) and a quasilikelihood approach called active set method (ASM) with Newton-Raphson estimation in GENLINMIXED (Heck et al., 2012, p. 27). We included random intercept as the random effect and selected variance components as the covariance structure for the random coefficients. We selected the Satterthwaite approximation to the degrees of freedom that were used to compute significance tests for model parameters, as recommended for data with varying number of individuals across clusters (Heck et al., 2012, p. 147). In the generalised linear mixed models for categorical outcomes, we additionally selected a robust, more conservative approach to the calculation of the standard errors of regression coefficients to allow departures from normality. Conventional single-level logistic regression models were built with the IBM SPSS NOMREG procedure for multinomial outcomes and with the IBM SPSS GENLIN procedure for dichotomous outcomes. Both procedures employ maximum likelihood estimation (Heck et al., 2012, p. 27).

For all outcome variables, we fitted first an intermediate model that included the primary predictor of our interest and then a final model that was adjusted for relevant covariates. As we used sites as observational units, independent variables included in the models were summarised to the site level to reflect site-level properties. The summarising concerned the following individual-level variables: physical work, a habit of eating at the worksite cafeteria, wish for support in healthy eating/physical activity, and the completion of the questionnaire both pre and post intervention. The summarising involved computing the proportion of individuals with the desired characteristic (e.g., physical work) per site and timepoint, and assigning the resulting values to the individual respondents of the corresponding site and time. The summarised variables were additionally grand-mean centred within the dataset that was included in the analysis by subtracting the overall sample mean from the site-level value. Grand-mean-centring recentres the site's standing on the variable against the sample mean and facilitates the interpretation of the coefficients of model parameters (Heck et al., 2012, p. 21).

Results

Table S5. Reasons for never performing restorative movements or using available exercise equipment.

Reason	% Pre	% Post	Difference in percentage points (post – pre)
for never performing movements	(n=203)	(n=139)	
Has not occurred to me	46	35	-11
Have no time	37	31	-6
Don't remember	23	35	12
Have no space	17	13	-4
Feel embarrassed	10	17	7
Don't want	9	11	2
Don't know how	3	3	0
Another reason[1]	2	6	4
for never using available exercise equipment	(n=210)	(n=230)	
Have no time	36	29	-7
Has not occurred to me	33	29	-4
Don't remember	30	35	5
Don't want	12	9	-3
Feel embarrassed	11	13	2
The equipment is not easily accessible	4	7	3
Don't know where the equipment is	3	1	-2
Don't know how	2	2	0
Another reason[2]	7	9	2

[1]e.g., move at home, no need, medical reason, physical work, work clothes.

[2]e.g., no need, medical reason, move after work, use breaks for eating, use break exercise application, prefer moving without equipment, laziness, heavy work clothing, pregnancy, does not feel good/useful, work community's objection, don't get around to using the equipment alone.

References

Heck, R. H., Scott, T. L., & Tabata, L. N. (2021). *Multilevel and longitudinal modeling with IBM SPSS* (Third edition). Routledge.

Heck, R. H., Thomas, S. L., & Tabata, L. N. (2012). *Multilevel modeling of categorical outcomes using IBM SPSS*. Routledge.

Lindström, J., Aittola, K., Pölönen, A., Hemiö, K., Ahonen, K., Karhunen, L., Männikkö, R., Siljamäki-Ojansuu, U., Tilles-Tirkkonen, T., Virtanen, E., Pihlajamäki, J., & Schwab, U. (2021). Formation and Validation of the Healthy Diet Index (HDI) for Evaluation of Diet Quality in Healthcare. *International Journal of Environmental Research and Public Health*, *18*(5), 2362. https://doi.org/10.3390/ijerph18052362
